# Molecular insight on the role of the phosphoinositide PIP_3_ in regulating the protein kinases Akt, PDK1, and BTK

**DOI:** 10.1042/BST20253059

**Published:** 2025-07-04

**Authors:** Alexandria L. Shaw, John E. Burke

**Affiliations:** 1 Department of Biochemistry and Molecular Biology, The University of British Columbia, Vancouver, British Columbia, V6T 1Z3, Canada; 2 Department of Biochemistry and Microbiology, University of Victoria, Victoria, BC, V8W 2Y2, Canada; 3 University of Victoria Genome BC Proteomics Centre, Victoria, BC, Canada

**Keywords:** cryo-electron microscopy, hydrogen-deuterium exchange mass spectrometry, kinases, PDK1, proto-oncogene proteins c-akt, structural biology

## Abstract

Protein kinases are master regulators of myriad processes in eukaryotic cells playing critical roles in growth, metabolism, cellular differentiation, and motility. A subclass of protein kinases is regulated by their ability to be localized and activated by the phosphoinositide phosphatidylinositol (3,4,5)-trisphosphate (PIP_3_). This includes multiple members of the AGC and TEC family kinases, which contain PIP_3_ binding pleckstrin homology (PH) domains. It has been postulated that they can be activated by PIP_3_-mediated disruption of autoinhibitory interactions between their kinase domains and PH domains. There has been considerable controversy based on differing molecular models for how these kinases are regulated by lipid binding and post-translational modifications. This review focuses on understanding the molecular underpinnings for how the PH domains of these enzymes regulate kinase activity and what role PIP_3_ plays in pathway activation. A specific focus is on the integration of experimental data derived from X-ray crystallography, cryo-electron microscopy, and hydrogen deuterium exchange mass spectrometry along with recent advances in artifical intelligence enabled protein modeling. The main lipid-binding enzymes described are the AGC protein kinases 3-phosphoinositide-dependent kinase (PDK1) and Akt, and the TEC family kinase, Bruton’s agammaglobulinemia tyrosine kinase (BTK).

## Introduction

The phosphorylation of proteins and peptides at the Ser, Thr, and Tyr amino acids is essential in almost all eukaryotic cellular processes. This is catalyzed by the transfer of the terminal phosphate from ATP by the action of protein kinases, with dysregulation of kinase activity being causative of many human diseases. This has made them important targets in pharmaceutical development with >30 kinase inhibitors being clinically approved for a wide variety of diseases [1–3]. The human kinome is extensive with >530 different kinases, representing ~2% of the protein coding genes in the human genome [4]. These can be classified into Ser/Thr kinases, Tyr kinases, and pseudokinases [5]. Many mechanisms have evolved to tightly control the activity of kinases, making sure that they are turned on in the correct cellular context. This is crucial for the fidelity of kinases involved in signaling cascades, with many requiring multiple activating inputs for full activation. Kinases have an inherent specificity for their substrates encoded by their kinase domains [6,7], but multiple additional factors can control activity including scaffolding, cellular localization, protein–protein interactions, metabolites, and post-translational modifications [8,9]. One of the regulatory mechanisms that can control kinase activity and localization is the targeted binding to lipid phosphoinositides, frequently mediated by lipid-binding domains. This review will explore how lipid phosphoinositides can regulate different kinases’ activity, with a specific focus on the molecular basis for how lipid-binding domains alter kinase activity by an analysis of previous biochemical/structural data in combination with recent advances in AlphaFold3-enabled protein modeling [10].

### General structural features of protein kinase regulation

Defining the basis for how lipid-binding domains alter kinase activity requires discussion of the molecular mechanisms controlling kinase activation. The first protein kinase structure of the enzyme protein kinase A (PKA) was solved ~35 years ago using X-ray crystallography [11], followed by extensive studies using X-ray, NMR, and cryo-EM with now more than half of all human kinase domains being structurally characterized, in a variety of active and inactive states [1]. This has allowed for the identification of multiple conserved features found in almost all active conformations of protein kinases and the divergent mechanisms by which protein kinases can be inactivated. Kinases often follow the Anna Karenina principle: while their active conformations are structurally similar featuring a conserved catalytic architecture necessary for function, their inactive conformations are diverse. This principle suggests that activation requires a conserved set of structural features, whereas inactivation results from unique structural disruptions. As a result, inactive kinases adopt a broad array of conformations, reflecting the many different ways activation can be perturbed [12].

**Figure 1:**
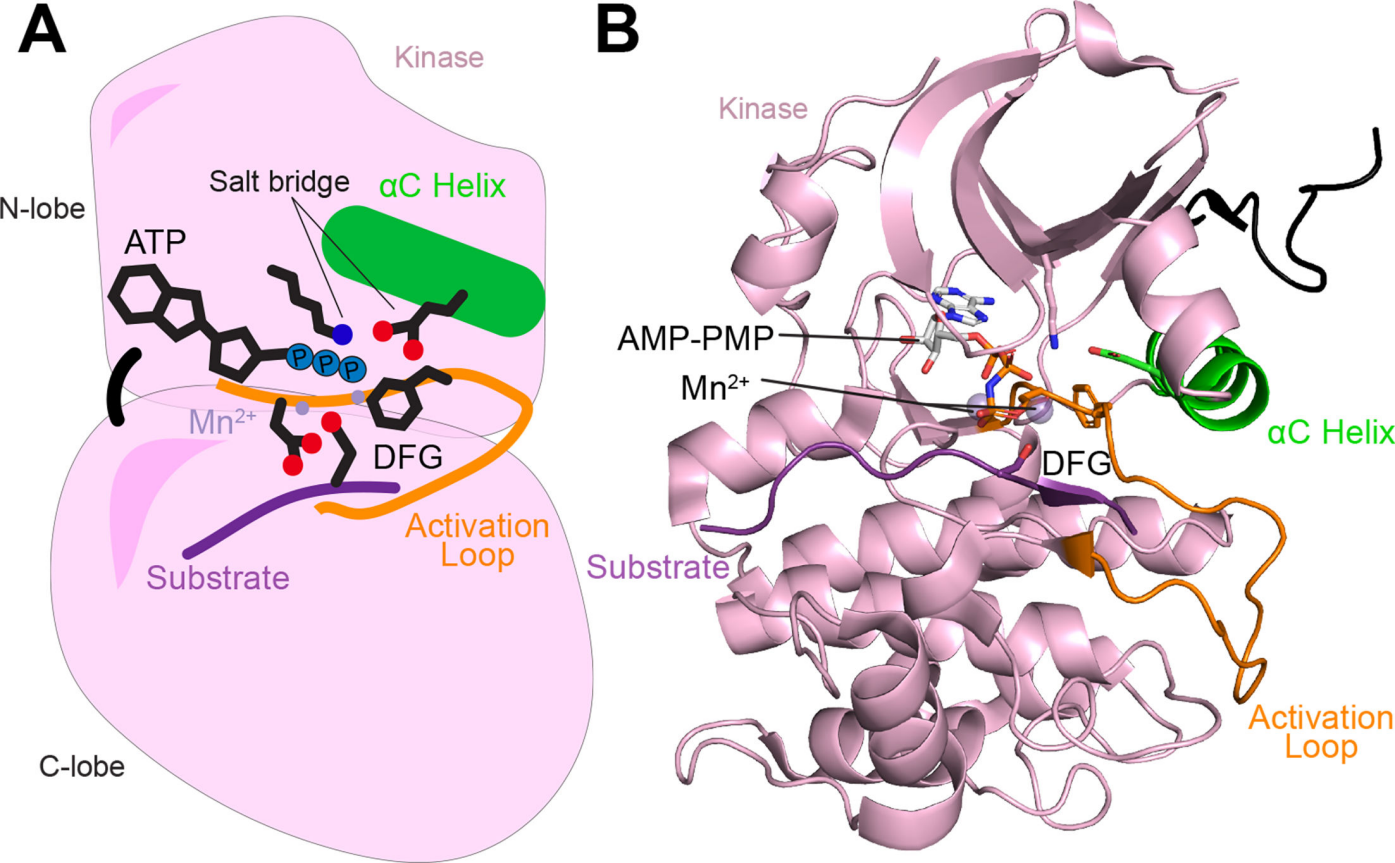
General conserved features of the active conformation of protein kinases Cartoon (**A**) and structural model (**B**) of the active conformation of a protein kinase based on the structure of active Akt (PDB:1O6K) bound to a non-hydrolyzable ATP mimetic adenylyl imidodiphosphate (AMP-PMP) [13]. The N-lobe, C-lobe, activation loop, αC helix, DFG motif, salt bridge, and peptide substrate are all indicated.

Due to space limitations, this review will only briefly survey these general mechanisms as they relate to lipid-regulated kinases, but readers are advised to consult previous reviews for a more in-depth analysis [1,8]. The kinase domain (KD) fold is a bi-lobal structure composed of an N-lobe and a C-lobe, with ATP binding at the cleft between these two lobes (Figure 1). In the active conformation, there are a set of hydrophobic interactions that occur in two spines that span the N- and C-lobes, with these being referred to as the regulatory and catalytic spines. Many aspects of kinase regulation are controlled by conformational changes between inactive states where the spines are disassembled. The region binding to ATP is often referred to as the hinge, with this being the connection point between the two lobes. The adenine ring of ATP forms part of the catalytic spine of hydrophobic interactions. Conserved features in protein kinases include the activation loop, containing residues that mediate binding to protein substrate. At the N-terminus of the activation loop, there is a strongly conserved DFG motif, with the aspartic acid playing an important role in co-ordinating the Mg^2+^ cofactor. The phenylalanine residue can exist in either a DFG-in or DFG-out conformation, with the DFG-in state being the active state. The change in orientation of this region of the activation loop plays a critical role in assembling the regulatory spine in the active conformation. Active kinase structures almost always contain a critical salt bridge from a glutamic acid in a conserved helix (αC helix) to a lysine in the β-sheet of the N-lobe. Together, these interactions are critical for orienting catalytic residues to maximize substrate catalysis. The activity of kinases can be modulated by modifying the conformation of the αC helix, mediated by post-translational modifications, protein–protein-binding partners, and intra-domain interactions.

This review focuses specifically on the structural basis of regulation of phosphoinositide-regulated kinases, specifically the AGC family kinases, Akt1/2 and 3-phosphoinositide-dependent kinase (PDK1), and the TEC family kinase, Bruton’s agammaglobulinemia tyrosine kinase (BTK). Specific regulatory features that control the activity of these kinases will be described in the following sections.

### AGC family kinases

#### PDK1

The AGC kinase family contains 63 serine/threonine kinases, including PDK1 and Akt/protein kinase B (PKB), which have highly conserved kinase domains [4]. PDK1 is a master regulator of cellular life [14]. When knocked out in mice, it results in early embryonic lethality, mainly driven by abnormalities in neuronal development [15]. PDK1 is described as a master regulator of kinase signaling, as it directly phosphorylates multiple AGC family kinases, including Akt/PKB [16], SGK, S6K [17], RSK [18], and PKC [19] kinase subfamilies. PDK1 is also able to phosphorylate and activate PKA [20]. However, PKA phosphorylation and activity are similar in both WT and PDK1 knockout embryonic stem cells [21], revealing that the ability to phosphorylate a substrate is not always directly linked to regulation. A unique regulatory feature of PDK1 is the presence of a PIFtide pocket within the N-lobe above the αC helix. This pocket is named for its ability to bind PDK1-interacting fragment (PIF) peptides, present in the hydrophobic motifs (HMs) of AGC substrate kinases [22,23]. This hydrophobic pocket plays a key allosteric role in regulating PDK1 activity, as it recruits downstream substrate kinases, and its occupancy by either substrates or small molecules leads to PDK1 activation [24].

**Figure 2:**
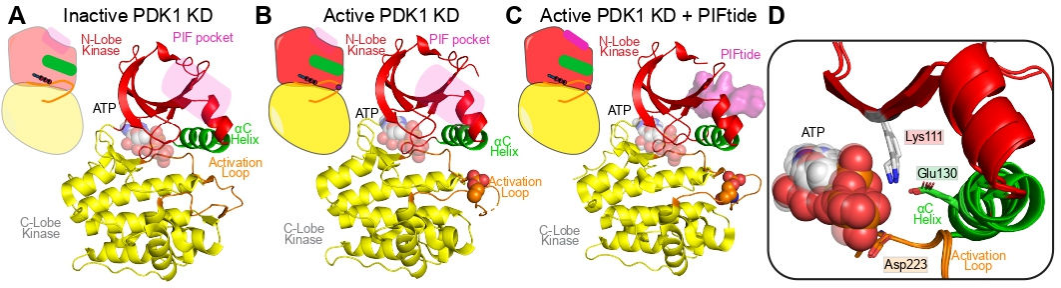
Structures of the PDK1 kinase domain in different states of activation Cartoon and structure of the (**A**) inactive PDK1 kinase domain (KD) with ATP (PDB:2BIY) [25], (**B**) active PDK1 KD (pS241) with ATP (PDB:1H1W) [26], and (**C**) active PDK1 KD (pS241) with ATP and PIFtide (PDB:4RRV) [27] with a (**D**) zoom-in of the overlayed catalytic cleft of structures from panel **A–C**, showing that it is in an active conformation regardless of activation state.

Crystal structures of PDK1’s kinase domain in different activation states show that the conformation of the catalytic cleft, irrespective of whether it is non-phosphorylated [25] or phosphorylated in the activation loop (S241) [26], or in the presence or absence of peptides bound to the PIF pocket [27], is in its active conformation (Figure 2A–D). The most well-studied PIF pocket binder is known as PIFtide, which is derived from the HM of PRK2. While the binding of the PIFtide to PDK1’s PIF pocket does not change the architecture of the catalytic cleft, there are several hydrogen bonds established upon PIFtide binding with the periphery of the αC helix, as well as the β-sheet packing against the β-strand containing the catalytic lysine, consistent with observed increases in the specific activity of PDK1 when PIFtides are bound to the pocket [22].

While many of PDK1’s substrates are other AGC family kinases, different inputs are required for the phosphorylation of different substrates [14]. For instance, substrates such as SGK, S6K, and PKC require docking of their C-terminal PIFtide to PDK1’s PIF pocket dependent on HM phosphorylation [28]. In contrast, PDK1-mediated phosphorylation of the Akt activation loop requires PI(3,4,5)P_3_ or PI(3,4)P_2_ [29,30], with data suggesting that Akt’s HM does not associate with PDK1’s PIF pocket [31]. Interestingly, PDK1 and its substrate Akt share pleckstrin homology (PH) domains with similar specificity toward PI(3,4)P_2_ and PI(3,4,5)P_3_ phosphoinositides. C-terminal to its kinase domain is PDK1’s PH domain that interacts with phosphoinositides [32]. Critical to the regulation of PDK1 is dimerization mediated by the PH domain [33], which occurs upon binding to cellular membranes and is promoted by anionic lipids [34]. However, controversy exists around the role of these PH domains in regulating kinase activity [14,35,36], and how phosphoinositides alter this activity for both PDK1 and Akt. This raises possible questions about whether the PH domain functions as part of an autoinhibitory regulatory mechanism or if phosphoinositides primarily serve to bring PDK1 substrates into its proximity at the plasma membrane.

**Figure 3:**
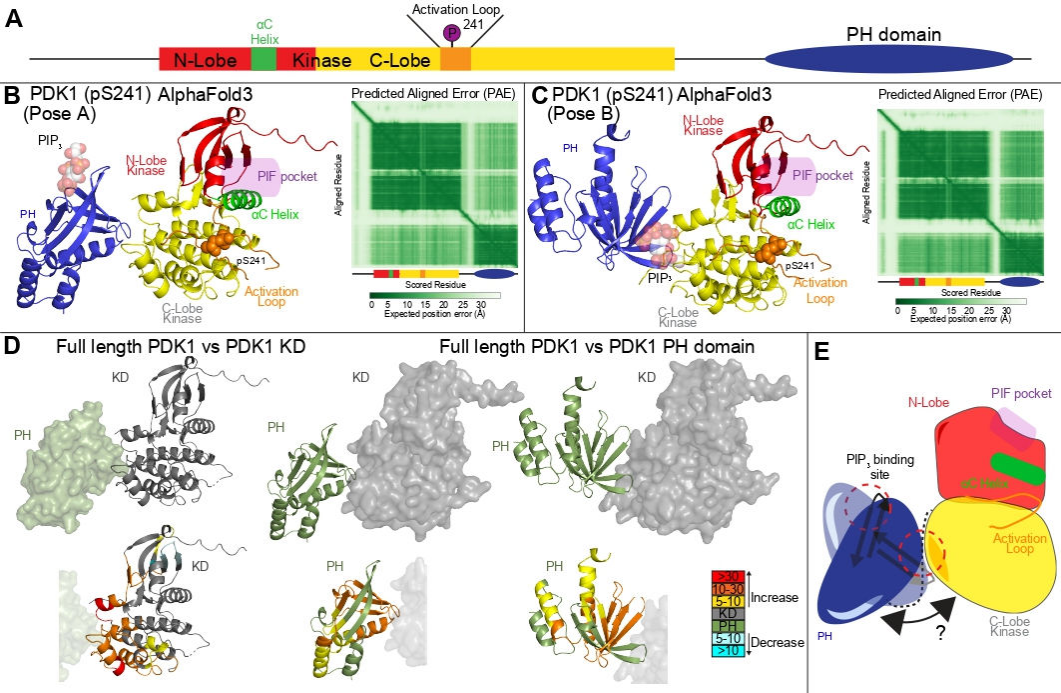
AlphaFold3 model of the conformational ensemble of full-length PDK1 (**A**) Domain schematic of PDK1 domain architecture highlighting key regulatory regions. (**B**) Most frequent orientation from the ensemble of AlphaFold3 [10] predictions (~70% of the total, from 10 different seeds, 50 total predictions) of full-length pS241 PDK1 with predicted local distance difference test (pLDDT) residues with scores < 50 removed. Experimental structure (PDB:1W1G) [32] containing PIP_3_ was aligned to the AlphaFold3 prediction, and PIP_3_ is overlaid to show the PIP_3_ binding site. (**C**) Second-most frequent orientation from the ensemble of AlphaFold3 predictions (~30% of the total, from 10 different seeds, 50 total predictions) with pLDDT< 50 removed. The predicted aligned error of the top-ranked model for **(B and C)** are shown to the right of each model. (**D**) Hydrogen deuterium exchange mass spectrometry (HDX-MS) data from [35] looking at HDX changes in the (left) kinase domain (KD) when comparing full-length PDK1 to the isolated KD, and changes in the (middle and right) PH domain when comparing full-length PDK1 to the isolated PH domain. Changes in the KD are mapped onto the AlphaFold3 prediction from panel **B**, and changes in the PH domain are mapped onto the AlphaFold3 predictions from panels **B** (middle) and **C** (right). (**E**) Cartoon model of the predicted ensemble of full-length PDK1.

There is currently no high-resolution structural information for how PDK1 and its PH domain interact; however, there are multiple hydrogen deuterium exchange mass spectrometry (HDX-MS) studies of full-length PDK1 and the kinase domain alone [35,36]. We used AlphaFold3 to examine this HDX-MS data compared to these AI models (Figure 3A–C). Modeling was done in the presence of activation loop phosphorylation (pS241) as this is likely the best mimic of the biological conformation of PDK1. This was done in the context of multiple unique seeds, to add a level of randomness into the resulting models. In all cases, the PH domain was predicted to form a PH-KD interface at the C-lobe (Figure 3B and C). Intriguingly, the ensemble of predictions has different orientations of the PH domain, with ~30% projecting the phosphatidylinositol (3,4,5)-trisphosphate (PIP_3_) binding site toward the kinase domain, and 70% with it oriented away from the kinase domain. The interface with the C-lobe of the kinase domain is supported by recent HDX-MS analysis [35] (Figure 3D), with HDX-MS changes in the PH domain likely suggestive of a conformational ensemble (Figure 3E), as neither pose can fully explain the HDX-MS differences. However, we did not observe the previously described interface of the PH domain with the linker in any searchers [36], with this linker having very limited secondary structure as determined by HDX-MS in full-length PDK1 [35]. This ensemble of predicted PDK1 structures with the C-lobe of the kinase domain interacting with the PH domain agrees with a hypothesized interface and is validated by mutational analysis [37].

The role of the PH domain in regulating kinase activity, as well as the role of phosphoinositides in regulating phosphorylation, is controversial. Intriguingly, substrate phosphorylation occurs under different regulatory mechanisms, as disruption of PH-mediated dimerization prevents Akt phosphorylation but does not block S6K phosphorylation [36]. Further experiments fully investigating the role of the PH and kinase interface in controlling PDK1 activity will allow for the proving or disproving of the possible regulatory role of the PH domain in PDK1 signaling.

#### Akt/PKB

**Figure 4:**
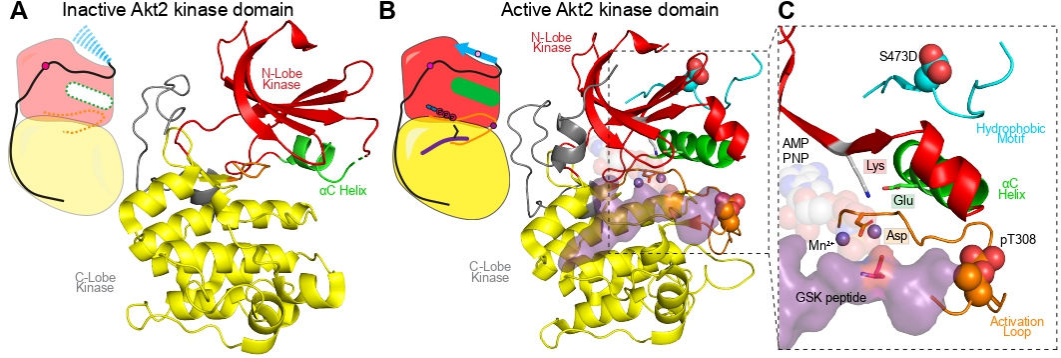
Structures of inactive and active conformations of Akt’s kinase domain (**A**) Inactive Akt kinase domain (PDB:1GZN) [41], with the activation loop being fully disordered, and the αC helix being partially disordered. (**B**) Active Akt kinase domain (pT308, S473D) with AMP-PNP and GSK peptide substrate. (PDB:1O6K) [13], with a (**C**) zoom-in on the active conformation of Akt’s catalytic cleft.

Akt (also referred to as protein kinase B, PKB) is a master regulator of cellular growth, metabolism, survival, and proliferation [38,39]. There are three isoforms of Akt (Akt1, Akt2, and Akt3), which are all activated downstream of phosphatidylinositol 3-kinase and remain in their inactive conformation in the absence of pro-growth signals. Akt activation occurs downstream of the second-most frequently mutated protein in human cancers (*PIK3CA*) and contains an activating oncogenic mutant (E17K) found primarily in breast and endometrial cancers. This makes it a promising therapeutic target, with the ATP-competitive inhibitor Capivasertib being FDA-approved for the treatment of hormone receptor-positive advanced breast cancer [40]. Like PDK1, Akt has a bi-lobal kinase domain housing similar catalytic machinery (Figure 4A and B). Unlike PDK1, Akt requires phosphorylation at two sites to adopt an active conformation, one in the activation loop (T308/309) by PDK1 [42,43] and the other in its C-terminal HM (S473/474) by mTORC2 [44] (Figure 4B and C). In the absence of these phosphorylation events, both the activation loop and HM are unstructured [41], along with a substantial region of the αC helix. Phosphorylation at the turn motif (T450/451) also occurs co-translationally to ensure correct folding of the bi-lobal kinase domain [45]. Unlike PDK1, Akt/PKB has no PIF pocket for their substrates to bind. Instead, its own phosphorylated HM binds the ‘PIF pocket’ (mediated by pS473/474), which, alongside activation loop phosphorylation at T308/309, drives the catalytic cleft to adopt an active conformation [13,41] through stabilization of the αC helix. To achieve full activation, Akt binds PIP_3_ or phosphatidylinositol (3,4)-bisphosphate (PIP_2_) at the plasma membrane through its PH domain [46]. However, whether PIP_3_/PIP_2_ binding is required once Akt is phosphorylated is still controversial. While kinase activity assays still show activation of fully phosphorylated Akt upon binding to PIP_3_ membranes, the activity in the absence of membranes is still orders of magnitude higher than unphosphorylated Akt1 [47,48].

**Figure 5:**
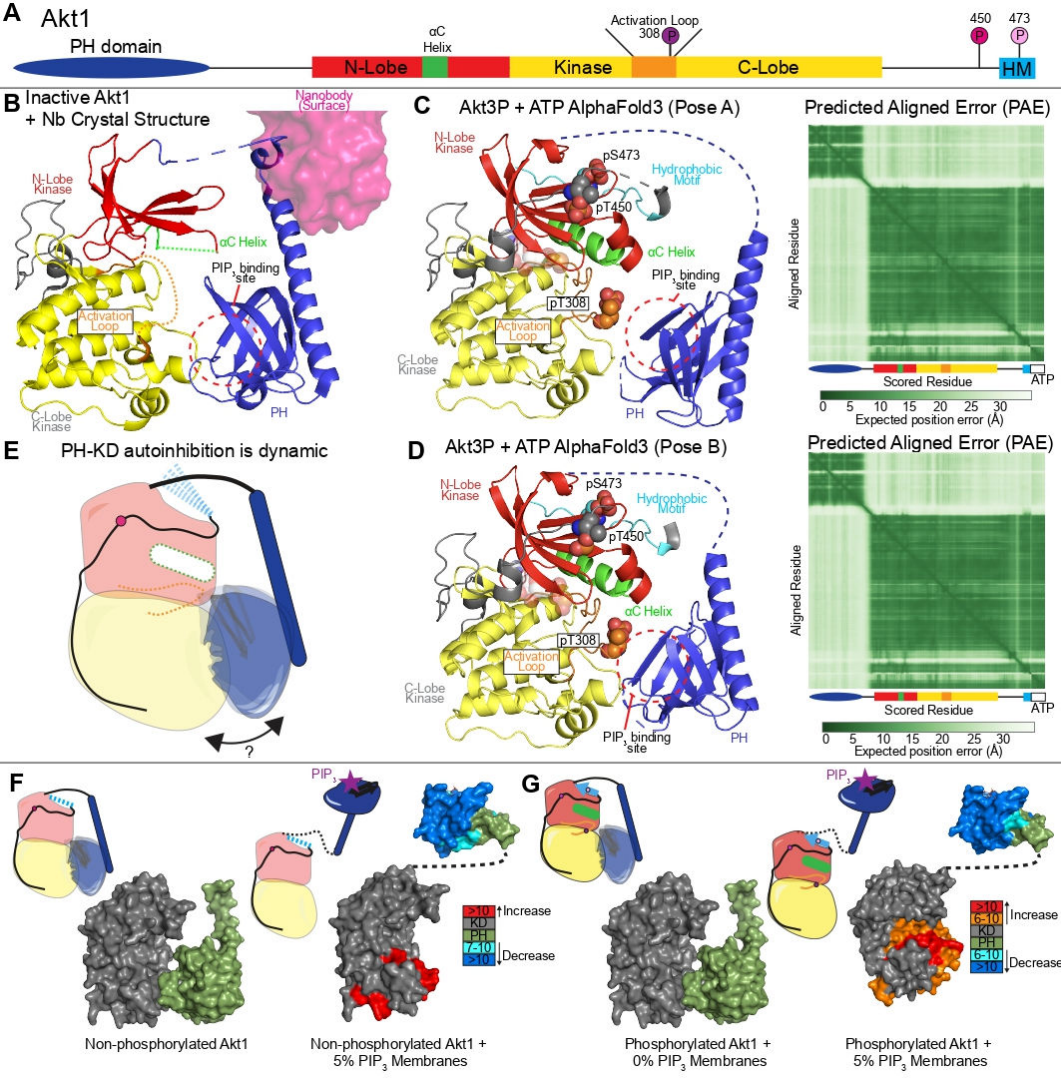
Autoinhibitory interface of Akt’s PH domain (**A**) Domain schematic of Akt domain architecture highlighting key regulatory regions. (**B**) Structure of inactive Akt with stabilizing nanobody (PDB:7APJ) [47]. (**C**) AlphaFold3 prediction of full-length tris-phosphorylated (pT308, pT450, pS473) Akt1 bound to ATP using five independent seeds (total 25 predictions), which predicted two unique orientations of the PH domain relative to the KD. Shown in **C** is the highest frequency model (Left) representing ~60% of the predictions with pLDDT< 60 removed. (Right) Predicted aligned error (PAE) plot of the AlphaFold3 prediction. (**D**) (Left) AlphaFold3 predictions representing ~40% of the predictions with pLDDT< 60 removed. (Right) PAE plot of the AlphaFold3 prediction. This shows a slight reorientation of the PH domain, but with the PIP_3_ binding interface still being occluded by the C-lobe of the kinase domain. (**E**) Cartoon representation of dynamic PH-KD autoinhibitory interface. (**F and G**) Hydrogen deuterium exchange mass spectrometry data from [47,48] comparing changes in monophosphorylated (pT450) (**F**) and phosphorylated (pT450, pT308, pS473) Akt1 (**G**) upon binding to 5% PIP_3_ membranes. There is an increase in deuterium exchange in the C-lobe at the proposed PH-KD interface when the PH domain is bound to PIP_3_ (as seen by a decrease in deuterium exchange in the PH domain). The structure of non-phosphorylated and phosphorylated with 0% PIP_3_ membranes (PDB:7APJ) represents the autoinhibited form in the absence of PIP_3_. Changes are mapped onto a molecular model consisting of (**F**) PDB:1UNQ [49] [PH bound to Ins(1,3,4,5)P_4_] and PDB:7APJ (isolated KD) or (**G**) PDB:4EKK [50] (active Akt1 KD, pT308, S473D) and PDB:1UNQ [PH bound to Ins(1,3,4,5)P4] .

To understand the mechanism of how phosphoinositides activate Akt requires the understanding of how the PH domain interacts with the rest of the enzyme. N-terminal to the kinase domain is a PH domain (Figure 5A). While it is agreed upon that it forms an autoinhibitory interface with the kinase domain, the role it plays in inhibiting kinase activity following Akt phosphorylation is still contentious. The most controversial aspect being how Akt phosphorylates substrates that are distant from any membrane compartment that does not contain PI(3,4)P_2_ or PIP_3_, with there being two competing models: one where Akt is only active when phosphoinositides are present, and one where once phosphorylated, Akt can diffuse to its targets in the absence of lipids. Critical to testing this model is a rigorous evaluation of the molecular interactions that phosphoinositide lipids regulate, specifically the interface between the PH and kinase domains.

The first molecular insight into mapping the PH domain autoinhibitory interface was from the structure of Akt1 in complex with the allosteric inhibitor MK-2206 [51] that showed that the PIP-binding site in the PH domain was sequestered at the bi-lobal interface of Akt. However, it remained unknown if this allosteric compound locked the PH domain in a non-native inhibitory conformation. Years later, the X-ray crystallography structure of an engineered human Akt1 construct containing a shorter PH-kinase linker derived from *Danio rerio* bound to a stabilizing nanobody was solved, revealing an interface between the kinase domain’s C-lobe and PH domain with >500 Å of buried surface area [47] (Figure 5B). AlphaFold3 predictions showed a slightly different orientation of the PH domain compared with either of these models (Figure 5C–E). Across all three models, the PH domain engages primarily with the C-lobe of the kinase domain, forming an interface that occludes the PIP_3_ binding pocket of the PH domain. While the general surface of the PH-kinase interaction is conserved across all three, the MK-2206-bound conformation exhibits the most deviation, where the PH domain is rotated and displaced relative to its position in the nanobody-stabilized and AlphaFold3-predicted structures. This unique orientation may reflect an inhibitor-induced conformational state that is either transient or less accessible under physiological conditions. Together, these structures suggest a model where the PH-KD autoinhibitory interface samples multiple conformations.

This is intriguing as it poses the question of whether the autoinhibitory PH-kinase interface can be maintained with and without regulatory phosphorylation, especially as the T308 site is located proximal to the interface with the PH domain. Work using protein semi-synthesis showed that phosphorylation of S473 relieves Akt autoinhibition through loss of an interaction with R144 in the PH-KD linker. Additionally, mutational analysis of R86 in the PH domain demonstrated decreased activity, suggesting it plays a role in establishing an autoinhibited Akt conformation [52]. Non-canonical HM phosphorylation events at S447 and T479 have also been proposed to relieve autoinhibition through a unique mechanism in the context of semisynthetic Akt [53]. However, HDX-MS studies argue that an autoinhibitory interface with the PH domain can be maintained in the T308/S473 phosphorylated state, as HDX-MS of a fully phosphorylated (T450, T308, S473) engineered Akt1 construct binding to 5% PIP_3_ membranes showed changes consistent with breaking of the PH-kinase interface [47]. This included increases in exchange in the KD’s C-lobe and a decrease in deuterium exchange in the PH domain consistent with the removal of the PH domain caused by an interaction with PIP_3_, with this occurring for both fully phosphorylated and monophosphorylated (T450) Akt1 (Figure 5 F and G). Interestingly, HDX-MS has shown a weakening of the PH-kinase autoinhibitory interface for both unphosphorylated and fully phosphorylated Akt1 binding to ATP-competitive inhibitors [54]. It is important to note that in both situations, the protein is either in the context of a truncated PH-kinase linker or protein semi-synthesis constructs that lack the critical T450 phosphosite. Therefore, fully resolving these conflicting biophysical results would require testing the activity and dynamics of both phosphorylated and non-phosphorylated states in as close to a biological mimic as possible using full-length Akt. It is possible that there are pools of Akt substrates that require phosphoinositides to both co-localize and maximally activate Akt activity, while other substrates can be phosphorylated by phosphorylated Akt once it is detached from membrane surfaces. This is consistent with recent work that showed differential phosphorylation of Akt substrates by distinct phospho-proteoforms, where different patterns of phosphorylation favored some Akt substrates over others [55].

### TEC family kinases

#### BTK

**Figure 6:**
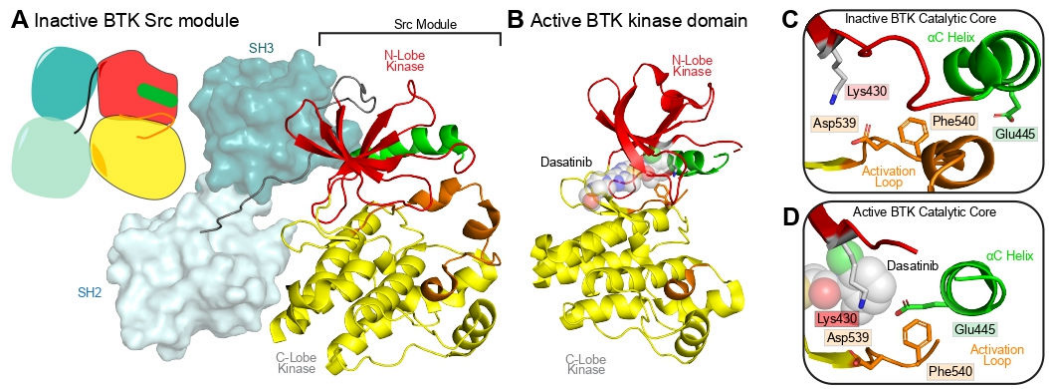
Structure of the Src module of BTK in its inactive state, and the kinase domain in its active state bound to Dasatinib (**A**) Cartoon and structure of the inactive BTK Src module (PDB:4XI2) [58], with the kinase domain shown in cartoon, and the SH2 and SH3 domains shown as surfaces. The αC helix is shown in green, with the activation loop colored orange. (**B**) Structure of the active conformation of BTK in complex with DFG-in inhibitor Dasatinib (PDB: 3K54) [59]. (**C and D**) Zoom-in views of the catalytic core in the (**C**) inactive (panel A) and (**D**) active (panel B) conformation of the catalytic cleft. The salt bridge between the N-lobe lysine and αC helix glutamic acid is broken in the inactive conformation.

BTK is a non-receptor tyrosine kinase in the TEC family kinase that plays a critical role in signal propagation downstream of the B-cell receptor, which is essential for B-cell development, differentiation, and signaling [56]. BTK is a therapeutic target in B-cell malignancies and autoimmune diseases, with multiple small-molecule inhibitors being FDA approved [57]. Inactivating mutations in BTK are causative of X‐linked agammaglobulinemia, leading to a significant decrease in mature B cells and frequent bacterial and viral infections. Structurally, BTK is regulated by its canonical Src module, consisting of a Src homology 2 (SH2), Src homology 3 (SH3), and kinase domain. The packing of the SH2 and SH3 domains against its SH3-kinase linker leads to the BTK kinase domain adopting an inactive conformation, with the αC helix rotated into an inactive state (Figure 6A - C) [58]. This inactive conformation of the Src module of BTK is similar to those of Src family kinases [60,61]. Disruption of the SH2 and SH3 interfaces leads to BTK’s kinase domain establishing its active conformation, where BTK can then become phosphorylated at Y551 in the activation loop by Src family kinases [62]. The active conformation of BTK bound to an ATP-competitive inhibitor is shown in Figure 6B - D[59], with the difference in conformation between inactive and active highlighted in Figure 6C and D.

**Figure 7:**
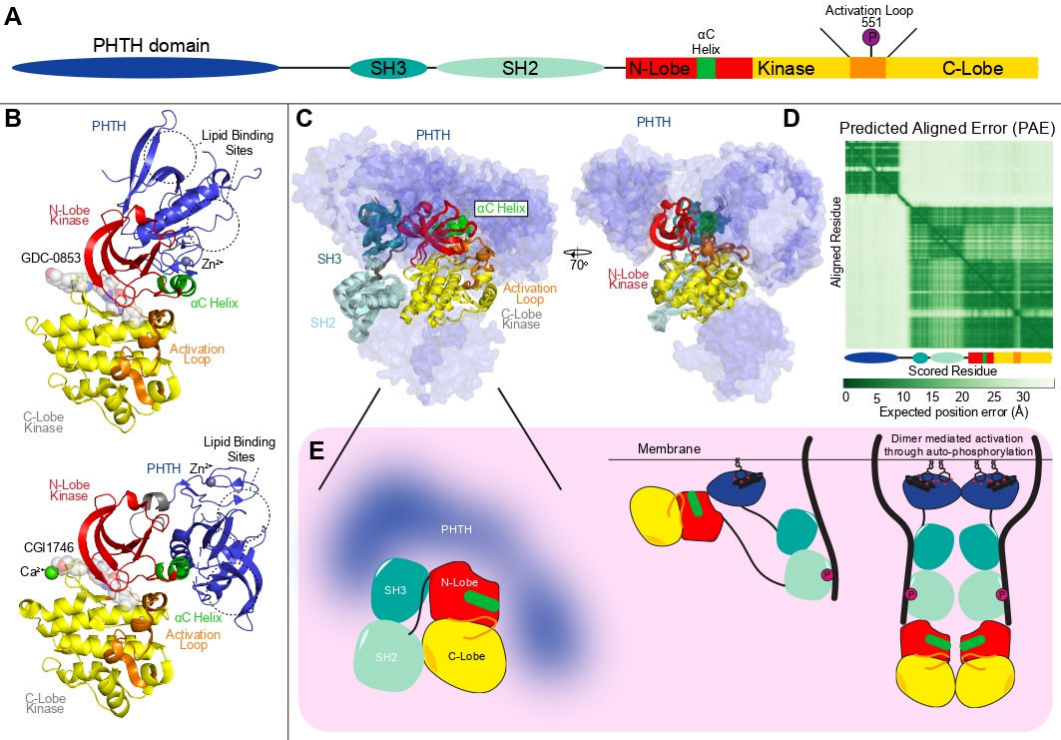
Conformational dynamics of full-length BTK and model of BTK activation (**A**) Domain schematic of BTK domain architecture highlighting key regulatory regions. (**B**) Structures of BTK PHTH-KD constructs lacking the SH2 and SH3 domains. (Top) BTK PHTH domain connected to KD with a glycine linker with GDC-0853 (PDB:8S93) [63]. (Bottom) BTK PHTH domain connected to KD with a SH2 (residues 384–393) linker with CGI1746 (PDB:4Y93) [58]. (**C**) Representation of five different seeds using AlphaFold3 with pY551 full length BTK, with 25 total predicted models. There is limited consensus on the orientation of the PHTH domain, with the 25 poses of the PHTH shown in surface, highlighting the dynamic nature relative to the Src module. This is consistent with recent low-resolution cryo-EM data, showing multiple possible orientations [63]. (**D**) Representative predicted aligned error (PAE) plot of the top scoring full-length phosphorylated BTK AlphaFold3 prediction, showing high error of relative position of the PHTH domain to the Src module. (**E**) Model of activation of BTK at the plasma membrane downstream of PIP_3_ and activated receptors.

N-terminal to the Src module is a PH domain and a Tec homology domain (defined as the PHTH module) (Figure 7A). However, the exact role of how these domains mediate BTK regulation is still not fully understood. The PH domain binds phosphatidylinositol (3,4,5)-trisphosphate (PIP_3_) [64], with PIP_3_ binding being essential for the membrane recruitment of BTK and its subsequent activation. PIP_3_ binding results in dimerization of the PH domain and clustering of full-length BTK [65]. PH domain-mediated dimerization can also be promoted by soluble inositol hexakisphosphate (IP_6_) [58] and is proposed to mediate activation by promoting trans-autophosphorylation of Y551. The role of the PHTH domains in regulating BTK in its inactive state remains unresolved. Crystal structures of full-length BTK show no clear density for the PHTH module, and low-resolution cryo-EM maps show multiple conformations of the PHTH relative to the Src module [63]. AlphaFold3 modeling using multiple seeds of full-length BTK (multiple seeds increases randomness) showed the PHTH domains sampling a large set of conformations, with limited high-confidence interactions occurring between these domains and the rest of the Src module (Figure 7C and D). HDX-MS analysis of full-length BTK versus the Src module of BTK revealed multiple regions with differences in exchange in multiple regions of the kinase domain, with these likely driven by HDX-MS sampling the conformational ensemble of PHTH conformations [66,67].

It has been proposed that the PH domain can bind to a partially active conformation of BTK, once the SH2 and SH3 domains are disengaged from the kinase domain. Structural insight into this has been provided by X-ray crystallography analysis of two constructs where the kinase domain is fused to the PHTH with different short linkers in the absence of the SH2+SH3 domains [58,63] (Figure 7B and C). In these structures, the PHTH domain was in an orientation incompatible with the presence of the SH2/SH3 domains. The use of different linkers revealed distinct orientations of the PHTH domain relative to the kinase domain, with both forming an interface with the N-lobe. This interface is likely dynamic and may serve to prevent full activation at low levels of PIP_3_ by preventing BTK trans-autophosphorylation. Upon increased PIP_3_ levels, the PHTH domain mediates dimerization, and BTK adopts a fully activated state (Figure 7E).

### Disease-linked mutations in PH domains of PIP_3_-regulated kinases

While the PH domains of Akt, PDK1, and BTK play important roles in regulating their activity, as discussed throughout the review, the occurrence of gain-of-function mutations at PH-KD interfaces is not uniformly shared. The most frequently observed oncogenic Akt mutation is E17K in Akt1, which is at the autoinhibitory interface between the kinase and PH domains. Its oncogenic mechanism is thought to involve enhanced phosphoinositide binding and increased membrane localization, without directly affecting kinase domain activity. However, this model remains under debate [52,68,69]. Equivalent mutations have not been reported in Akt2 or Akt3, which is likely due to their more restricted tissue distributions. Akt1 is ubiquitously expressed, whereas Akt2 is enriched in insulin-responsive tissues, and Akt3 is primarily found in brain, testis, and lung tissues. PDK1, although a central kinase in the PI3K/Akt pathway, has no reported oncogenic mutations. In BTK, disease-linked loss-of-function mutations are associated with X-linked agammaglobulinemia (XLA) [70,71]. Mutations in XLA patients contain missense, nonsense, deletions, and insertions, with some of these missense mutations clustering around the phosphoinositide-binding pocket of the PH domain, leading to reduced lipid binding and impaired BTK membrane localization [72], resulting in a loss of function. However, a distinct gain-of-function mutation, E41K, which is similar to that of Akt1’s E17K mutation, enhances phosphoinositide binding and drives constitutive membrane association [73,74]. It remains to be seen if this mutant has any influence on any PH domain autoinhibition of BTK.

## Summary

Recent years have seen significant advances in our structural understanding of how lipid-binding PH domains regulate multiple different families of protein kinases. These studies have revealed both significant differences in the mechanisms of how phosphoinositide lipids can control kinase activity. Better tools to measure kinase signaling in different intracellular compartments, along with better tools for tracking and manipulating phosphoinositides, will allow for the full testing of different molecular models. Critical to the field is interpreting structural studies within the specific experimental caveats required to capture unique structural states that may exist in their biological context in a large conformational ensemble. There has been significant clinical progress being made in inhibiting PIP_3_-regulated protein kinases, including multiple clinically approved inhibitors. It will be essential to fully work out the molecular basis of their activation in their native biological contexts, as this may be important to interpret the action of different ATP-competitive and allosteric inhibitors.

**Perspectives**

- Lipid-regulated protein kinases are master regulators of many essential functions, with multiple inhibitors now in the clinic for multiple human cancers. As both allosteric and ATP-competitive inhibitors lead to allosteric conformational changes, it is essential to understand the molecular basis of their regulation.
- Extensive biochemical, biophysical, and structural analysis of lipid-regulated protein kinases has revealed mechanisms of autoinhibition driven by lipid-binding domains, as well as how they are activated by phosphoinositides. Differing molecular models for the role of lipid-binding domains in autoinhibition and activation have been proposed.
- Inhibitors toward lipid-regulated kinases have made significant clinical impact. However, it is essential to understand the full complement of mechanisms that control lipid-regulated kinase activation. Continued investigation in more native biological contexts will be needed to fully define the mechanism of regulation.

## Abbreviations

BCR: B-cell receptor
BTK: Bruton’s agammaglobulinemia tyrosine kinase
HDX-MS: hydrogen deuterium exchange mass spectrometry
HM: hydrophobic motif
PAE: Predicted aligned error
PDK1: 3-phosphoinositide dependent kinase
PH: pleckstrin homology
PIF: PDK1 interacting fragment
PIP_2_: phosphatidylinositol (3,4)-bisphosphate
PIP_3_: phosphatidylinositol (3,4,5)-trisphosphate
PKA: protein kinase A
SH2: Src homology 2
SH3: Src homology 3
XLA: X-linked agammaglobulinemia
cryo-EM: cryogenic electron microscopy
